# Demographic, clinical characteristics and cardiovascular disease burden in a Portuguese cohort of older chronic kidney disease patients

**DOI:** 10.1590/2175-8239-JBN-2018-0120

**Published:** 2019-01-10

**Authors:** Josefina Maria Sousa Santos Lascasas, Isabel Fonseca, Jorge Malheiro, Sofia Santos, Andreia Campos, Ana Castro, Carla Moreira, Sofia Correia, Idalina Beirão, Luísa Lobato, António Cabrita

**Affiliations:** 1 Centro Hospitalar do Porto, Hospital de Santo António, Departamento de Nefrologia, Porto, Portugal.; 2 Universidade do Porto, Instituto de Ciências Biomédicas Abel Salazar, Unidade Multidisciplinar de Investigação Biomédica, Porto, Portugal.; 3 Universidade do Porto, Instituto de Saúde Pública, Porto, Portugal.

**Keywords:** Renal Insufficiency, Chronic, Cardiovascular Diseases, Aged, Insuficiência Renal Crônica, Doenças Cardiovasculares, Idoso

## Abstract

**Introduction:**

Chronic kidney disease (CKD) is an independent risk factor for several unfavorable outcomes including cardiovascular disease (CVD), particularly in the elderly, who represent the most rapidly growing segment of the end-stage kidney disease (ESKD) population. Portugal has the highest European unadjusted incidence and prevalence rates of ESKD. In 2012, we started to follow a cohort of elderly CKD patients, we describe their baseline characteristics, risk profile, and cardiovascular disease burden.

**Methods:**

All CKD patients aged 65 years and older referred to our department during 2012 were enrolled. Baseline data included: demographic, CKD stage, medication, comorbid conditions. Estimated glomerular filtration rate (eGFR) was calculated by the CKD-EPI formula.

**Results:**

A total of 416 patients, 50% referred by primary care physicians, aged 77 ± 7 years, 52% male, with a median eGFR of 32 mL/min/1.73m^2^ participated in the study. Fifty percent had diabetes (DM), 85% dyslipidemia, 96% hypertension; 26% were current/former smokers, and 24% had a body mass index > 30 kg/m^2^. The prevalence of CVD was 62% and higher in stage 4-5 patients; in diabetics, it gradually increased with CKD progression (stage 3a < stage 3b < stage 4-5) (39, 58, 82%; *p* < 0.001).

**Conclusions:**

At baseline, our CKD elderly cohort had a higher burden of CVD. The prevalence of CVD was greater than in other European CKD cohorts. Lower level of eGFR was associated with a greater burden of CVD and was more pronounced in diabetics, highlighting the importance of strategically targeting cardiovascular risk reduction in these patients.

## RESUMO

**Introdução:**

Doença renal crônica (DRC) é fator de risco independente para vários desfechos desfavoráveis, incluindo doença cardiovascular (DCV), particularmente em idosos, o segmento de crescimento mais rápido da população com doença renal terminal (DRT). Portugal tem a maior incidência europeia não-ajustada e a maior prevalência de DRT. Neste artigo caracterizamos uma coorte de idosos com DRC, referenciados para a nefrologia, com particular ênfase para o risco e carga de doença cardiovascular.

**Métodos:**

Foram incluídos todos os pacientes com DRC com 65 anos ou mais encaminhados ao nosso departamento em 2012. Os dados basais incluíram: demografia, estágio da DRC, medicação e comorbidades. A taxa de filtração glomerular (TFGe) foi calculada pela fórmula CKD-EPI.

**Resultados:**

Metade dos 416 pacientes incluídos foram encaminhados por médicos da atenção primária; sua idade era 77 ± 7 anos; 52% eram homens; a TFGe mediana era de 32 mL /min/1,73 m^2^. Metade tinha diabetes (DM), 85% dislipidemia, 96% hipertensão; 26% eram fumantes atuais/ antigos; 24% tinham índice de massa corporal > 30 kg/m^2^. A prevalência de DCV foi de 62%, sendo maior entre pacientes nos estágios 4-5; em diabéticos, aumentou gradualmente com a progressão da DRC (estágio 3a < estágio 3b < estágio 4-5) (39%, 58%, 82%; *p* < 0,001).

**Conclusões:**

A coorte de idosos com DRC apresentava inicialmente maior carga de DCV. A prevalência de DCV foi maior que em outras coortes europeias com DRC. Níveis menores de TFGe foram associados a carga maior de DCV e foram mais pronunciados entre diabéticos, destacando a importância de objetivar estrategicamente a redução do risco cardiovascular nesses pacientes.

## INTRODUCTION

Chronic kidney disease (CKD) has emerged as a serious public health problem, as shown by the increase in overall and cardiovascular mortality and the growing incidence and prevalence of end-stage kidney disease (ESKD), with patients requiring renal replacement therapy and leading to very high health-care costs^1^. Parallel to this, the prevalence of CKD is higher in older people, and patients over 65 years of age represent the most rapidly growing segment of the ESKD population in wealthier countries^2^^,^ 3.

Portugal has the highest unadjusted incidence and prevalence of ESKD among European countries^4^ and 62.3% of the incident dialysis patients in 2016 were over 65 years with a mean age of prevalent patients of 67 years^5^.

CKD is associated with increased prevalence of both traditional (e.g., hypertension) and nontraditional cardiovascular risk factors (e.g., proteinuria, elevated uric acid levels, hyperhomocysteinemia), and predisposing factors to microvascular disease (e.g., inflammation, inflammation, abnormal calcium-phosphate homeostasis)^6^^,^ 7, with studies confirming that in the elderly, even in early CKD stages, cardiovascular mortality outweighs the risk of progression to ESKD^8^.

In 2012, we started to follow a cohort of elderly patients until the occurrence of the first event (ESKD or death)^9^. In this report we describe their baseline demographic and clinical characteristics, with particular emphasis for the cardiovascular disease burden, to define improved strategies of care.

## PATIENTS AND METHODS

### STUDY DESIGN AND POPULATION

This study included consecutive CKD (non-dialyzed and non-transplanted) patients aged ≥ 65 years, newly referred to our outpatient department in Centro Hospitalar do Porto (CHP), within January 1, 2012 and December 31, 2012. CHP is a tertiary-care hospital that serves a diverse population of 500 000 inhabitants in the North region of the country.

Data collection was conducted by nephrologists using electronic case report forms. Reporting of cardiovascular disease was based on both the patients’ self report and review of their medical records by trained staff on the same date of the baseline interview. The study was performed in accordance with the Declaration of Helsinki and approved by the Institutional Review Board of CHP.

Baseline date included sex, age, body mass index (BMI), CKD stage, proteinuria, medication, and comorbid conditions, such as diabetes, dyslipidemia, hypertension, smoking status, and cardiovascular disease. Cardiovascular disease was defined as the history of at least one of the following: cardiac disease, cerebrovascular disease, and peripheral vascular disease. Cardiac disease was defined as the history of coronary artery disease, congestive heart failure, and severe valvular heart disease with or without valvular replacement. Criteria for the diagnosis of coronary artery disease included previous myocardial infarction, angina pectoris, coronary artery bypass grafting, or percutaneous transluminal coronary angioplasty with or without stent implantation. Cerebrovascular disease included previous transient ischemic attack, stroke, or cerebral hemorrhage. Peripheral artery disease was defined as the presence of intermittent claudication, need of peripheral revascularization, or amputation.

All diabetic patients met the classification criteria established by the American Diabetes Association. Hypertension was considered if the patient had systolic blood pressure (BP) > 140 mmHg or diastolic BP > 90 mmHg or need for antihypertensive drugs. Dyslipidemia included total serum cholesterol > 200 mg/dL, or triglycerides > 150 mg/dL, or high-density lipoprotein (HDL) cholesterol < 40 mg/dL in males and < 48 mg/dL in females, or low-density lipoprotein (LDL) cholesterol > 100 mg/dL, or need for lipid-lowering drugs.

Glomerular filtration rate was estimated (eGFR) using the Chronic Kidney Disease Epidemiology (CKD-EPI) 2009 creatinine equation^10^. Etiological diagnosis of CKD was based on the patient’s history, kidney ultrasound, and kidney biopsy, when available. Blood and urine routine measurements were collected: hemoglobin, serum albumin, urea nitrogen, creatinine, calcium, phosphorus, intact parathyroid hormone (PTH), glucose, hemoglobin A1c, uric acid, lipid profile, iron, unsaturated iron binding capacity, ferritin, and urine protein-to-creatinine ratio (uPCr) in spot urine sample.

Cognitive status was evaluated and screened using the Mini Mental State Examination (MMSE)^11^, classified as cognitive impairment if the score was 23 or lower. Functional dependency was defined as the requirement of assistance in the activities of daily living, and classified as totally dependent, partially dependent, and autonomous.

A modified version of the Charlson comorbidity index (mCCI)^12^ i.e., subject’s age and presence or absence of kidney disease was excluded, was calculate to assess severity of comorbidities.

### STATISTICAL ANALYSIS

Baseline characteristics are described using mean ± standard deviation or median with interquartile ranges for continuous variables, while categorical data are presented as numbers and percentages. Cardiovascular disease burden was compared between CKD stages by Chi-squared test for trend for categorical variables. Statistical analyses were performed using SPSS version 22.0. P-value < 0.05 was considered statistically significant.

## RESULTS

### BASELINE DEMOGRAPHIC AND CLINICAL CHARACTERISTICS

From a total of 848 patients newly referred to our Nephrology department during 2012, 416 of them were 65 years and older. Of these, all were Caucasians, 52% were male, with a mean age of 77 years, and 36% of them were aged 80 years or more. About 50% (n = 206) of the patients were diabetic. The majority (85%) of the study population came from urban areas.

Their baseline characteristics, divided by gender and by the presence or absence of diabetes are summarized in Table 1. Fifty percent of the patients were referred by primary care physicians. At baseline, they had a median serum creatinine of 1.6 mg/dL and a median eGFR of 32mL/min per 1.73 m^2.^ The most frequent etiologies of renal disease were ischemic nephropathy (n = 159; 38%) and diabetic nephropathy (n = 106; 26 %); unknown causes of renal disease were 55 (13%). Only 4% (n = 17) of the patients had a renal biopsy.

**Table 1:** Baseline characteristics stratified by gender and the presence and absence of diabetes *mellitus*

		Male n = 218		Female n= 198	
	Total n = 416	Diabetics n = 112	Non-Diabetics n = 106	Diabetics n = 94	Non-Diabetics n = 104
Age (years), mean;SD	76.9 ± 7.4	75.2 ± 7.1	77.6 ± 7.6	75.9 ± 7.2	78.8 ± 7.2
Age ≥ 80 years, n (%)	149 (36)	28 (25%)	43 (41%)	30 (32%)	49 (47%)
eGFR EPI (ml/min/1.73 m^2^), median;IQR	32 [23-42]	30 [22-39]	27 [21-37]	33 [25-43]	34 [27-47]
Serum creatinine (mg/dL), median;IQR	1.6 [1.3-2.0]	1.7 [1.3-2.1]	1.7 [1.4-2.2]	1.4 [ 1.2-1.9]	1.4 [ 1.1-1.8]
CKD Stage, n (%)					
Stage 1	6 (1.4)	0 (0.0)	0 (0.0)	3 (3.2)	3 (2.9)
Stage 2	33 (7.9)	11 (9.8)	8 (7.5)	8 (8.5)	6 (5.8)
Stage 3a	46 (11.0)	9 (8.0)	10 (9.4)	9 (9.6)	18 (17.3)
Stage 3b	139 (33.4)	36 (32.1)	24 (22.6)	37 (39.4)	42 (40.4)
Stage 4	158 (38.0)	43 (38.4)	50 (47.2)	33 (35.1)	32 (30.8)
Stage 5	34 (8.2)	13 (11.6)	14 (13.2)	4 (4.3)	3 (2.9)
Referral, n (%)					
Primary care	207 (49.8)	54 (48.2)	50 (47.2)	49 (52.1)	54(51.9)
Hospital appointment	191 (45.9)	54 (48.2)	52 (49.1)	41 (43.6)	44 (42.3)
Other	18 (4.3)	4 (3.6)	4 (3.8)	4 (4.3)	6 (5.8)
Renal disease etiology, n (%)					
Ischemic nephropathy	159 (38.2)	36 (32.1)	52 (49.1)	22 (23.4)	49 (47.1)
Diabetic nephropathy	106 (25.5)	62 (55.4)	0 (0.0)	44 (46.8)	0 (0.0)
Glomerulonephritis	16 (3.8)	1 (0.9)	8 (7.5)	3 (3.2)	4 (3.8)
Polycystic kidney disease	7 (1.7)	1 (0.9)	4 (3.8)	1 (1.1)	1 (1.0)
Miscellaneous	73 (17.5)	9 (8.0)	25 (23.6)	13 (13.8)	26 (25.0)
Unknown	55 (13.2)	3 (2.7)	17 (16.0)	11 (11.7)	24 (23.1)
mCCI score ≥ 5, n(%)	105 (25.2)	55 (49.1)	14(13.2)	31(32.9)	5(4.8)
BMI (kg/m^2^), mean;SD	27.3 ± 4.8	27.1 ± 4.9	25.7 ± 4.1	29.5±5.5	27.0 ± 4.6
BMI > 30 (kg/m^2^), n (%)	101 (24.4)	19 (16.9)	13 (12.3)	43 (45.7)	23 (22.1)
BMI > 25 to ≤ 30 (kg/m^2^), n (%)	174 (42.0)	60 (53.6)	43 (40.5)	31 (33.0)	44 (42.3)
BMI ≤ 25 (kg/m^2^), n (%)	139 (33.6)	33 (29.5)	50 (47.2)	20 (21.3)	37 (35.6)
Current smokers, n (%)	22 (5.3)	8 (7.1)	10 (9.4)	2 (2.1)	2 (1.9)
Former smokers, n (%)	87(20.9)	44 (39.3)	40 (37.8)	2 (2.1)	1 (1.0)
Never smokers, n (%)	307(73.8)	60 (53.6)	56 (52.8)	90 (95.8)	101 (97.1)
SBP (mm Hg), mean;SD	140.9 ± 24.1	142.7 ± 22.6	139.4 ± 22.1	140.9 ± 26.7	140.5 ± 25.2
DBP (mm Hg), mean;SD	71.7 ± 12.5	71.8 ± 11.4	72.5 ± 12.4	71.3 ± 12.5	71.3 ± 13.9
MAP (mmHg), mean;SD	94.8 ± 14.7	95.9 ± 13.5	93.7 ± 13.5	94.7 ± 16.2	94.7 ± 15.9
BP < 130/80 mmHg, n (%)	130 (31.3)	30 (26.8)	31 (29.2)	32 (34.0)	37 (35.6)
BP < 140/90 mmHg, n (%)	198 (47.6)	48 (42.9)	49 (46.2)	49 (52.1)	52 (50.0)
Antihypertensive ≥ 3, n (%)	58 (13.9)	20 (17.9)	16 (15.1)	10 (10.6)	12 (11.5)
Renin-angiotensin blockade, n (%)	293 (70.4)	86 (76.8)	72 (67.9)	65 (69.1)	70 (67.3)
Dyslipidemia, n (%)	354 (85)	104 (92.9)	81 (76.4)	85 (90.4)	84 (80.8)
Lipid-lowering medication, n (%)	248 (59.6)	73 (65.2)	60 (56.6)	54 (57.4)	61 (58.7)
Albumin (g/dL)	4.09 ±0.50	4.08 ±0.49	4.20±0.46	3.98 ± 0.55	4.08 ± 0.49
Uric acid (mg/dL), mean;SD	7.3 ± 2.2	7.3 ± 2.1	7.3 ± 2.2	7.1 ± 2.3	7.2 ± 2.4
Total cholesterol (mg/dL),mean;SD	180 ± 49	176 ± 46	173 ± 45	178 ± 50	192 ± 53
HDL (mg/dL), mean;SD	48 ± 14	45 ± 13	50 ± 16	44 ± 12	52 ± 15
LDL (mg/dL), mean;SD	105 ± 40	102 ± 38	101 ± 37	104 ± 42	113 ± 42
Hemoglobin (g/dL), mean;SD	12.1 ± 1.8	12.4 ± 1.7	12.7 ± 2.1	11.3 ± 1.5	11.9 ± 1.5
Hemoglobin < 11g /dL, n (%)	110 (26.4)	23 (20.5)	26 (24.5)	36 (38.3)	25 (24.0)
TSAT (%), mean;SD	22 ± 10	22 ± 10	25 ± 11	18 ± 9	23 ± 11
Ferritin (ng/mL), mean;SD	245 ± 251	246 ± 269	286 ± 263	188 ± 187	271 ± 261
iPTH (pg/mL), mean;SD	125.0 ± 90.3	120.5± 101.0	134.6 ± 81.7	114.5 ± 74.5	132.2± 98.6
Calcium (mg/dL), mean;SD	2.37 ± 0.18	2.36 ± 0.16	2.37 ± 0.16	2.38 ± 0.22	2.39 ± 0.20
Phosphate (mg/dL), mean;SD	1.14 ± 0.22	1.13 ± 0.25	1.06 ± 0.23	1.18 ± 0.21	1.17 ± 0.22
uPCr (g/g), mean;SD	1.10 ± 2.20	1.33 ± 2.27	0.72 ± 1.48	1.29 ± 2.38	0.99 ± 2.46

Continuous variables are presented as mean ± standard deviation or medians and interquartile ranges when appropriate. Categorical data are presented as numbers (n) of patients and percentages (%).The number of missing values was < 1% for all parameters except to UPCr (20%). eGFR, estimated glomerular filtration rate; mCCI, modified Charlson comorbidity index; BMI, body mass index; SBP, Systolic blood pressure; DBP, Diastolic blood pressure; MAP, Mean Arterial Pressure; HDL, high density lipoprotein; LDL, low density lipoprotein; TSAT, transferrin saturation; iPTH, intact parathyroid hormone; uPCr, urinary protein-to-creatinine ratio.

Most of patients were nonsmokers (n = 307; 74%). The proportion of current or former smokers was highest in men with (n = 52; 46%) and without diabetes (n = 50; 47%). Overall, 24% (n = 101) of enrolled patients were obese (BMI > 30 kg/m^2^). The BMI ranged from a mean value of 25.7 ± 4.1 kg/m^2^ in men without diabetes to 29.5 ± 5.5 kg/m^2^ in women with diabetes.

About 96% (n = 400) of the patients presented hypertension, with a mean BP of 141/72 mmHg. In approximately 30% of the patients it was < 130/80 mmHg and in approximately 50% it was < 140/90 mmHg; men with diabetes were the group with worst BP control (BP goal < 130/80 mmHg). About 50% (n = 207) of the patients were receiving two or more antihypertensive drugs (excluding diuretics), and 14% (n = 58) were under three or more antihypertensive drugs. Inhibitors of the renin angiotensin system were the drugs most frequently used (n = 293; 70% of the patients): angiotensin-converting enzyme (iECA) inhibitors in 33% (n = 137), angiotensin II receptor blockers (ARB) in 41% (n = 172), and combined iECA and ARB in 4% (n = 16) of the patients. The use of these agents was more frequent in men with diabetes (77%). About 71% (n = 296) of the patients were on diuretics.

Dyslipidemia was present in 85% of the patients (n = 354) and 60% (n = 248) were under lipid-lowering medication. Dyslipidemia was more prevalent in patients with diabetes (n = 189; 92%).

An active or previous malignancy was present in 15% (n = 62) of patients. About 25% (n = 105) of the patients had a high comorbidity index (mCCI score ≥ 5), particularly men with diabetes (n = 55; 49%).

About 50% (n = 206) of the patients were diabetic, but in only 51% (n = 106) of them, diabetic nephropathy was considered the etiology of renal disease; 48% of those patients with etiologies of CKD other than diabetic nephropathy had diabetes. When we analyzed baseline characteristics in patients with diabetes separately for patients with and without diabetic nephropathy in comparison to patients without diabetes (Supplementary Table S1), clinical and demographic data were very similar in the three groups, with the exception of a higher systolic BP, the use of more antihypertensive drugs, higher prevalence of dyslipidemia, and higher proteinuria level in those with presumed diabetic nephropathy.

Regarding functional dependency, 6% (n = 25) of the patients were totally dependent, and 38% (n = 158) were partially dependent with no difference between the groups. Cognitive impairment was present in 12% (n = 50) of the patients, with no difference between the groups.

Globally, eGFR was slightly lower in men, particularly those without diabetes. The mean uPCr rate was 1.1, higher in patients with diabetes when compared to patients without diabetes.

Most patients had a hemoglobin level ≥ 11 g/dL (n = 306; 74%), and the percentage of patients with transferrin saturation < 20% was 38% (n = 158), and ferritin level < 100 was 25% (n = 104), respectively. Only 5% (n = 21) of the total cohort was receiving erythropoiesis stimulating agents (ESA). The percentage of patients on oral and IV iron supplementation were 10% (n = 42) and 0.7% (n = 3), respectively.

Intact parathyroid hormone was elevated in 81% (n = 337) of the patients, despite good control of calcium-phosphorus levels. The percentage of patients being treated with vitamin D supplementation and phosphate binders was 7% (n = 28) and 4% (n = 17), respectively.

### BASELINE CARDIOVASCULAR PREVALENCE

Cardiovascular disease was present in 62% (n = 256) of the patients: coronary artery disease in 25% (n = 103), cerebrovascular disease in 24% (n = 100), and peripheral vascular disease in 19% (n = 77), respectively (Table 2).

**Table 2:** Cardiovascular disease burden stratified by gender and presence and absence of diabetes *mellitus*

	Total n = 416	Male n = 218		Female n= 198	
		Diabetics n = 112	Non-Diabetics n = 106	Diabetics n = 94	Non-Diabetics n = 104
Cardiovascular disease*, n; (%)	256 (62)	74 (66)	65 (61)	60 (64)	57 (55)
Cardiac disease, n;(%)	282 (68)	78 (70)	62 (58)	85 (90)	57 (55)
Coronary artery disease	103 (25)	34 (30)	25 (24)	30 (32)	14 (13)
Congestive heart failure	164 (39)	42 (38)	34 (32)	50 (53)	38 (37)
Severe valvular heart disease	15 (4)	2 (2)	3 (3)	5 (5)	5 (5)
Cerebrovascular disease, n; (%)	100 (24)	35 (31)	25 (24)	18 (19)	22 (21)
Peripheral artery disease, n; (%)	77 (19)	33 (29)	18 (17)	16 (17)	10 (10)

* Cardiovascular disease includes all patients with one or more of the following: cardiac disease, cerebrovascular and peripheral vascular disease. Categorical data are presented as numbers (n) of patients and percentages (%).

Coronary artery disease was present in 31% (n = 64) of the patients with diabetes, compared to 19% (n = 39) in patients without diabetes.

Previous cerebrovascular events were more frequent in men compared to women: 28% (n = 60) and 20% (n = 40), respectively; the prevalence was only slightly higher in patients with diabetes compared to patients without diabetes: 26% (n = 53) and 22% (n = 47), respectively.

Peripheral vascular disease was more prevalent in patients with diabetes compared to patients without diabetes, 24% (n = 49) and 13% (n = 28), respectively, and in men compared to women, 23% (n = 51) and 13% (n = 26), respectively.

**Figure 1:**
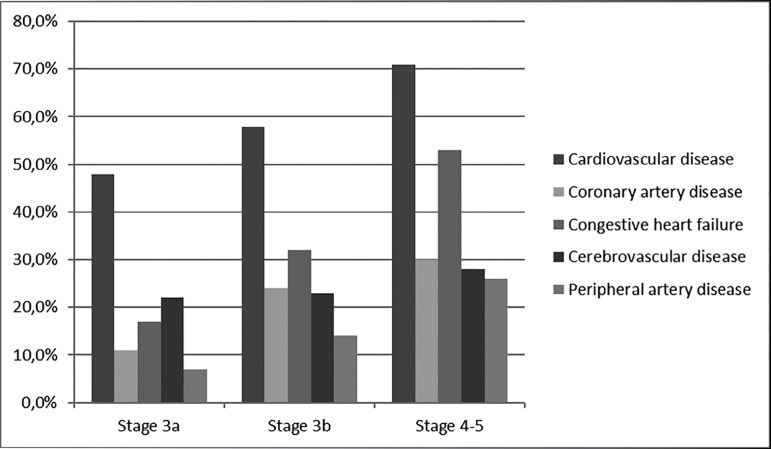
Prevalence (%) of individual causes of cardiovascular disease stratified by CKD stages.

Stratifying the CKD stages in 3a, 3b, and 4-5 the prevalence of coronary artery disease, congestive heart failure, and peripheral vascular disease were highest in stage 4-5 patients, gradually increasing with CKD progression (Figure 1). The cardiovascular disease burden associated with eGFR declining was more pronounced in patients with diabetes, compared to patients without diabetes (Table 3).

**Table 3:** Cardiovascular disease burden, stratiﬁed by CKD stages and presence and absence of diabetes *mellitus*

	Total n = 377				Diabetics n = 184				Non-Diabetics n = 193			
CKD Stage	3a n = 46	3b n = 139	3b n = 139	*p*	3a n = 18	3b n = 73	4-5 n = 93	*p*	3a n = 28	3b n = 66	4-5 n = 99	*p*
Cardiovascular disease*, n (%)	22 (48)	80 (58)	137 (71)	0.002	7 (39)	42 (58)	76 (82)	< 0.001	15 (54)	38 (58)	61 (62)	0.713
Cardiac disease, n(%) Coronary artery disease	5(11)	33 (24)	58 (30)	0.022	2 (11)	22 (30)	36 (39)	0.062	3 (11)	11 (17)	22 (22)	0.339
Congestive heart failure	8 (17)	45 (32)	102 (53)	< 0.001	2 (11)	26 (36)	59 (63)	< 0.001	6 (21)	19 (29)	43 (43)	0.040
Severe valvular heart disease	2 (4)	3 (2)	10 (5)	0.371	1 (6)	1 (1)	5 (5)	0.375	1 (4)	2 (3)	5 (5)	0.805
Cerebrovascular disease, n (%)	10 (22)	32 (23)	54 (28)	0.475	2 (11)	16 (22)	34 (37)	0.027	8 (29)	16 (24)	20 (20)	0.610
Peripheral artery disease, n(%)	3 (7)	20 (14)	49 (26)	0.003	3 (17)	13 (18)	30 (32)	0.071	0	7 (11)	19 (19)	0.022

* Cardiovascular disease includes all patients with one or more of the following: cardiac disease, cerebrovascular and peripheral vascular disease. Categorical data are presented as numbers (n) of patients and percentages (%). Cardiovascular disease burden was compared between CKD stages by Chi-squared test for trend for categorical variables. P-value < 0.05 was considered statistically significant.

## DISCUSSION

When we designed our longitudinal cohort study, the main objective was to identify the main predictors for CKD progression and death in elderly CKD patients referred to our outpatient department^9^. In this report we analyzed their baseline characteristics, cardiovascular risk profile, and cardiovascular disease prevalence.

A particular characteristic of this study is that all enrolled patients were newly referred to our nephrology department. This gives us information on the baseline characteristics of the patients before they receive specific attention from the nephrologist.

In our cohort, the two most frequent causes of CKD were ischemic and diabetic nephropathy, which are considered leading causes of CKD worldwide^13^^,^ 14, particularly in older patients. In 13% of the patients, CKD etiology was considered unknown. However, in the absence of specific diagnostic tests and given the low biopsy rate of 4%, the diagnostic certainty was low.

The prevalence of cardiovascular disease in our elderly CKD cohort was very high, present in 62% of the patients. Patients with CKD are more prone to develop cardiovascular disease^15^. Data available from several epidemiological studies revealed that cardiovascular events and cardiovascular mortality increased inversely with eGFR^16^^,^ 17. Conversely, cardiovascular disease is associated with increased risk of CKD progression^18^. Furthermore, the older the individual with CKD, the highest the risk of cardiovascular disease and mortality, and even more so if additional comorbid conditions including diabetes, hypertension, obesity, and other vascular risk factors are present^19^.

In our cohort, many established risk factors of cardiovascular disease showed a high prevalence; with an increase in the prevalence of cardiovascular disease with worse CKD stage (Table 3 and Figure 1). The cardiovascular disease burden associated with eGFR decline was pronounced in patients with diabetes (Table 3). The cardiovascular disease prevalence was higher than that reported from in European CKD cohorts (German GCKD^20^, Spanish MERENA^21^, Italian CARHES^22^), even when adjusting for age.

Although our study was not designed to identify risk factors for CKD and for cardiovascular disease, several characteristics of the patients provided indirect evidence for several predisposing factors.

First, the diabetes prevalence of 50% is almost twice as high as in the PREVADIAB^23^, a population-based study to evaluate the prevalence of diabetes in Portugal. Also, the prevalence of diabetes in our cohort was higher than that reported in other Europeans CKD cohorts (German GCKD^20^: 35%; Spanish MERENA^21^: 41%; Italian CARHES^22^: 28%), even when adjusting for age.

Among the traditional risk factors for CKD and for cardiovascular disease, the presence of hypertension in our cohort was almost universal (96%), without significant differences between diabetic and non-diabetic patients. In terms of hypertension control, blood pressure was < 130/80 mmHg in only approximately one-third of the patients. This gap between targets and clinical reality is consistent with other CKD cohort studies^20^^-^ 22, which illustrates the difficulties of blood pressure control in CKD, and a potential for improvement. A large percentage of patients were taking renin angiotensin system inhibitors (70%), particularly men with diabetes (77%). However, men with diabetes were the group with worst BP control, which may contribute to the role of male gender and diabetes in cardiovascular disease burden.

Current smoking was reported in 5% of the cohort, and former smoking in 21 % (Table 1), with a male preponderance. This is a lower prevalence than that reported in the other CKD cohorts 20 ^-^ 22 ^,^ 24.

Overall, 24% of enrolled patients were obese (BMI > 30 kg/m^2^), which is lower than the prevalence in other European CKD cohorts^20^^-^ 22. Therefore, obesity was not a major contributor to cardiovascular disease prevalence in our study group. Even so, obesity and overweight were more prevalent in diabetic patients.

Prediction of the renal and cardiovascular risks in people with CKD is likely to be improved by the incorporation of albuminuria into kidney disease staging^25^. The data in our study regarding proteinuria was similar to that reported in other European CKD cohorts^21^^,^ 22. On the other hand, the degree of proteinuria in our cohort is higher than other cohorts (CRIC)^24^, which may be related to the better BP control in those studies. The level of proteinuria was higher in patients with diabetes compared to patients without diabetes, confirming that proteinuria is a hallmark of diabetic nephropathy, but also an important contributor to the cardiovascular risk in diabetic patients.

Dyslipidemia is also a traditional cardiovascular risk factor that is frequently observed in CKD patients, with an increasing incidence with CKD progression^26^. Dyslipidemia was very prevalent in our cohort (85 %) and more prevalent in patients with diabetes, which reinforces the cardiovascular risk in those patients.

Anemia is also an associated factor of cardiovascular disease prevalence and mortality, and CKD progression^27^. The mean hemoglobin of the cohort was 12.1 g/dL, and the majority of the patients had hemoglobin greater than 11 g/dL (74%). Since only 5% of the patients in the total cohort were receiving ESA therapy, the relatively high hemoglobin of the group as a whole cannot be attributed to overtreatment with these agents. Only 10% of the patients was receiving iron therapies, but transferrin saturation < 20% was documented in 38% of the patients, reflecting insufficient treatment with iron before nephrology referral.

CKD-mineral bone disorder is a major contributor to vascular calcification and cardiovascular disease^28^ in CKD patients. Concerning calcium-phosphorus and PTH levels, an evident finding was that only 19% of the patients in our cohort had PTH levels within the recommended targets based on K-DOQI guidelines^29^, despite good control of calcium-phosphorus levels. A very low percentage of patients receiving vitamin D and its analogues was found, suggesting that a further management optimization for CKD-mineral bone disorder is needed.

The prevalence of diabetes as well as other risk factors for CKD may partly explain international variation in CKD prevalence. In a recent narrative review on the factors that potentially underlie observed international differences in CKD prevalence in the elderly within Europe^30^, the authors concluded that Portugal had the highest estimate of CKD prevalence, and the highest average score on CKD risk factors (i.e. diabetes mellitus, high blood pressure, physical inactivity, and salt intake).

The strengths of our study include the rigorous exploration of the first Portuguese CKD cohort with patients aged 65 years and over newly referred to a nephrology clinic. Furthermore, this was a representative group of patients, and knowing their baseline characteristics and cardiovascular morbidity will allow a better understanding of CKD epidemiology, nephrology referral policy in our geographical area, and the global approach to cardiovascular risk.

There are certain limitations to our research. First, this is a single-center study. Second, some misclassification bias maybe have been introduced based on the patients’ self-reporting of comorbidity. Finally, because this cohort only comprised patients attending the nephrology outpatient clinic, our results may not necessarily translate to CKD patients who are not referred to nephrologists.

## CONCLUSIONS

In summary, the characteristics of our referral CKD cohort demonstrated the heavy burden of cardiovascular risk profile and disease, and reflected an important role of several risk factors for kidney disease development. The prevalence of diabetes and cardiovascular disease is greater than other European CKD cohorts.

Lower eGFR level was associated with a greater burden of cardiovascular disease, highlighting the importance of strategically targeting cardiovascular risk reduction in these older patients. This is an important group of patients that should be characterized and understood; our results should improve management of these patients over time.

## SUPPLEMENTARY MATERIAL

The following online material is available for this article:

Table S1 - Comparison of patients with diabetes *mellitus* (DM) with and without presumed diabetic nephropathy (DN) and patients without diabetes mellitus
