# Supplementary material for: Demographic, clinical characteristics and cardiovascular disease burden in a Portuguese cohort of older chronic kidney disease patients
Source: J Bras Nefrol. 2019 Jan 10;41(1):29–37. doi: 10.1590/2175-8239-JBN-2018-0120 (PMC6534027; doi:10.1590/2175-8239-JBN-2018-0120)
Supplement: Supplementary file 1 [file 2175-8239-jbn-2018-0120-suppl01.pdf]

Supplementary Material to "Demographic, clinical characteristics and cardiovascular disease burden in a Portuguese cohort of older chronic kidney disease patients"

Table S1. Comparison of patients with diabetes mellitus (DM) with and without presumed diabetic nephropathy (DN) and patients without diabetes mellitus

	**No DM n=210**	**DM with DN n=106**	**DM without DN n=100**
Age (years), mean; SD	78.2 ± 7.4	74.6 ± 6.7	76.5 ± 7.4
Age ≥80 years, n (%)	92 (43.8)	22 (20.8)	36 (36.0)
Male, n (%)	106 (50.5)	62 (58.5)	50 (50.0)
Female, n (%)	104 (49.5)	44 (41.5)	50 (50.0)
eGFR EPI (ml/min/1.73 m2), median; IQR	30 [23-39]	33 [23-44]	32 [24-42]
Serum creatinine (mg/dL), median; IQR	1.6 [1.3-2.1]	1.3 [1.2-2.0]	1.5 [1.3-2.0]
**CKD Stage, n (%)**			
Stage 1	2 (1.0)	3 (2.8)	1 (1.0)
Stage 2	17 (8.1)	10 (9.4)	6 (6.0)
Stage 3a	23 (10.9)	11 (10.4)	12 (12.0)
Stage 3b	66 (31.4)	40 (37.7)	33 (33.0)
Stage 4	85 (40.5)	29 (27.4)	44 (44.0)
Stage 5	17 (8.1)	13 (12.3)	4 (4.0)
**Referral, n (%)**			
Primary care	103 (49.0)	53 (50.0)	50 (50.0)
Hospital appointment	98 (46.7)	51 (48.1)	45 (45.0)
Other	9 (4.3)	2 (1.9)	5 (5.0)
mCCI score ≥ 5, n(%)	19 (9.0)	44 (41.5)	42 (42.0)
BMI (kg/m2), mean; SD	26.2 ± 4.8	28.1 ± 5.1	28.3 ± 4.9
BMI >30 (kg/m²), n (%)	36 (17.1)	28 (26.4)	34 (34.0)
BMI >25 to ≤30 (kg/m²), n (%)	86 (41.0)	49 (46.2)	42 (42.0)
BMI ≤25 (kg/m2), n (%)	88 (41.9)	29 (27.4)	24 (24.0)
Current smokers, n (%)	12 (5.7)	5 (4.7)	5 (5.0)
Former smokers, n (%)	41(19.5)	23 (21.7)	23 (23.0)
Never smokers, n (%)	157(74.8)	78 (73.6)	72 (72.0)
SBP (mm Hg), mean; SD	139.9 ± 23.6	146.4 ± 20.9	137.1 ± 27.2
DBP (mm Hg), mean; SD	71.9 ± 13.1	73.0 ± 11.4	70.1 ± 12.3
MAP (mmHg), mean; SD	94.6 ±14.8	97.5 ± 13.0	92.4 ± 15.9
BP <130/80 mmHg, n (%)	68 (32.4)	19 (17.9)	43 (43.0)
BP <140/90 mmHg, n (%)	101 (48.1)	43 (40.6)	54 (54.0)
Antihypertensive ≥3, n (%)	28 (13.3)	21 (19.8)	9 (9.0)
Renin-angiotensin blockade, n (%)	142 (67.6)	81 (76.4)	70 (70.0)
Calcium channel blockers, n (%)	56 (26.6)	53 (50.0)	41 (41.0)
Beta blockers, n (%)	72 (34.3)	35 (33.0)	33 (33.0)
Diuretics, n (%)	140 (66.6)	77 (72.6)	79 (79.0)
Dyslipidemia, n (%)	165 (78.6)	104 (98.1)	85 (85.0)
Lipid-lowering medication, n (%)	121 (57.6)	65 (61.3)	62 (62.0)
Antiplatelet medication, n (%)	92 (43.8)	65 (61.3)	47 (47.0)
Albumin (g/dL)	4.09 ± 0.52	4.10 ± 0.49	4.09 ±0.48
Albumin <3.5 g/dL, n (%)	20 (9.5)	6 (5.6)	8 (8.0)
Uric acid (mg/dL), mean; SD	7.3 ± 2.3	7.0 ± 2.0	7.5 ± 2.4
Total cholesterol (mg/dL),mean; SD	183± 50	178 ± 51	176 ± 43
HDL (mg/dL), mean; SD	51 ± 15	44 ± 13	46 ± 13
LDL (mg/dL), mean; SD	107 ± 40	103 ± 44	102 ± 34
Hemoglobin (g/dL), mean; SD	12.3 ± 1.9	11.9 ± 1.7	11.8 ± 1.0
TSAT (%), mean; SD	24 ± 11	19 ± 9	21± 10
Ferritin (ng/mL), mean; SD	169 ± 261	184 ± 198	261 ± 270
uPCr (g/g), mean; SD	0.77 ± 1.98	1.79 ± 2.73	0.58 ± 1.2

Continuous variables are presented as mean ± standard deviation or medians and interquartile ranges when appropriate. Categorical data are presented as numbers (n) of patients and percentages (%). eGFR, estimated glomerular filtration rate; BMI, body mass index; SBP, Systolic blood pressure; DBP, Diastolic blood pressure; MAP, Mean Arterial Pressure; HDL, high density lipoprotein; LDL, low density lipoprotein; TSAT, transferrin saturation; uPCr, urinary protein-to-creatinine ratio
